# Diagnostic Challenges in Aspiration Pneumonia With Repeated Isolation of Rothia aeria: A Case Report

**DOI:** 10.7759/cureus.85824

**Published:** 2025-06-12

**Authors:** Toshiaki Motegi, Kenta Nakamura, Shunsaku Matsu, Kiyoshi Shibuya, Shigen Hayashi

**Affiliations:** 1 Respiratory Medicine, Tsukuba Medical Center Hospital, Tsukuba, JPN; 2 Respiratory Medicine, Ibaraki Seinan Medical Center Hospital, Sakai, JPN

**Keywords:** actinomyces infection, aspiration pneumonia, bacterial pneumonia, infection disease, rothia aeria

## Abstract

*Rothia aeria* (*R. aeria*) is a rare, facultatively aerobic Gram-positive bacillus in Actinomycetales. Although it is known as part of the normal oral flora, it has increasingly been reported as an opportunistic pathogen, particularly in immunocompromised patients. However, reports of respiratory infections due to *R. aeria* remain extremely rare. We report a case of an elderly woman with aspiration pneumonia in whom *R. aeria* was repeatedly isolated from sputum cultures. Branching Gram-positive bacilli were observed on Gram stain; however, these organisms were not found within neutrophils, suggesting a lack of active phagocytosis. The patient showed rapid clinical and radiological improvement following short-term treatment with meropenem. Post-treatment culture of bronchoalveolar lavage fluid was negative. Due to the minimal inflammatory response and lack of overt tissue destruction, the findings were interpreted as either transient colonization or mild infection. This case highlights the importance of comprehensive clinical judgment in evaluating the clinical significance of rare organisms isolated from non-sterile specimens rather than relying solely on culture results. Our case suggests that, even in relatively immunocompetent hosts, repeated detection of *R. aeria* in respiratory specimens warrants careful consideration of its pathogenic potential, particularly in the context of radiological changes and clinical response to treatment.

## Introduction

Pulmonary actinomycosis is an infection caused by actinomycetes, which are normally part of the oral flora and are mainly classified into the genera* Actinomyces* and* Nocardia*. *Actinomyces* grow under strictly anaerobic conditions, while *Nocardia* grow strictly under aerobic conditions [1]. Both cause chronic and slowly progressive infections characterized by non-specific clinical and radiological findings, which are often misdiagnosed as malignancy or other chronic pulmonary diseases. Symptoms include the gradual onset of fever, cough, and chest pain, with typical findings such as abscess formation, sinus tracts, and fibrosis. Diagnosis is usually based on culture and histological examination.

*Rothia* is a Gram-positive bacillus known to be part of the normal flora of the oral cavity and upper respiratory tract. It is facultatively aerobic and is phylogenetically related to* Actinomyces*, based on 16S ribosomal ribonucleic acid gene sequencing (16S rRNA sequencing). Among these, *Rothia aeria* (*R. aeria*), first isolated from air inside the Mir space station in 2004 [2], is an extremely rare opportunistic pathogen. It has been reported to cause infections such as endocarditis, sepsis, and pneumonia, particularly in immunocompromised individuals [3].

Aspiration pneumonia often involves the right lower lobe when patients are in a sitting or upright position; however, when patients eat while supine, involvement of the posterior segments of the upper lobes or the superior segments of the lower lobes, including the left upper lobe, can occur. Accurate species identification requires methods such as 16S rRNA sequencing or matrix-assisted laser desorption/ionization time-of-flight mass spectrometry (MALDI-TOF-MS).

We present a case of an elderly woman who developed aspiration pneumonia during hospitalization, with radiographic infiltrates predominantly in the left upper lobe. *R. aeria* was repeatedly isolated from high-quality sputum specimens. Although the clinical response to antimicrobial therapy was favorable, the absence of phagocytosed organisms on Gram stain and negative post-treatment bronchoalveolar lavage (BAL) cultures raised diagnostic uncertainty regarding whether this represented colonization or true infection.

Differentiating true infection from colonization remains a diagnostic challenge, particularly in immunocompetent patients without overt signs of inflammation. This case underscores the importance of comprehensive clinical judgment in evaluating the clinical significance of rare organisms isolated from non-sterile specimens.

## Case presentation

An elderly woman in her 80s was admitted to the orthopedic department for conservative treatment of a lumbar vertebral fracture. She remained on prolonged bed rest and had been eating in a supine position for approximately three weeks. On hospital day 20 (designated as day Y), she developed a fever of 38.0°C and bilateral pulmonary infiltrates, raising suspicion of aspiration pneumonia. She was referred to the respiratory department on the same day. Her medical history included hypertension and diabetes mellitus, both of which were well-controlled with medication. She was a farmer by occupation and had no recent history of dental procedures.

At the time of consultation, her body temperature was 37.8°C and her oxygen saturation was 92% on 1 L/min of oxygen via nasal cannula, indicating mild hypoxemia. Blood tests showed mild leukocytosis and elevated C-reactive protein (CRP) levels (Table 1).

**Table 1:** Blood Test Results at the Time of Referral and Serial Blood Test Results During Treatment. WBC: white blood cells; CRP: C-reactive protein; Y: the day the patient was referred to the respiratory department

Test	At referral (Y day)	Y+2 day	Y+5 day	Y+7 day	Y+10 day	Y+14 day
WBC (μL)	19410	15290	8170	6460	5960	4600
Neutrophil (%)	79.40	-	-	-	-	-
CRP (ng/mL)	33.38	34.27	17.26	6.35	3.44	0.97

**Figure 1:**
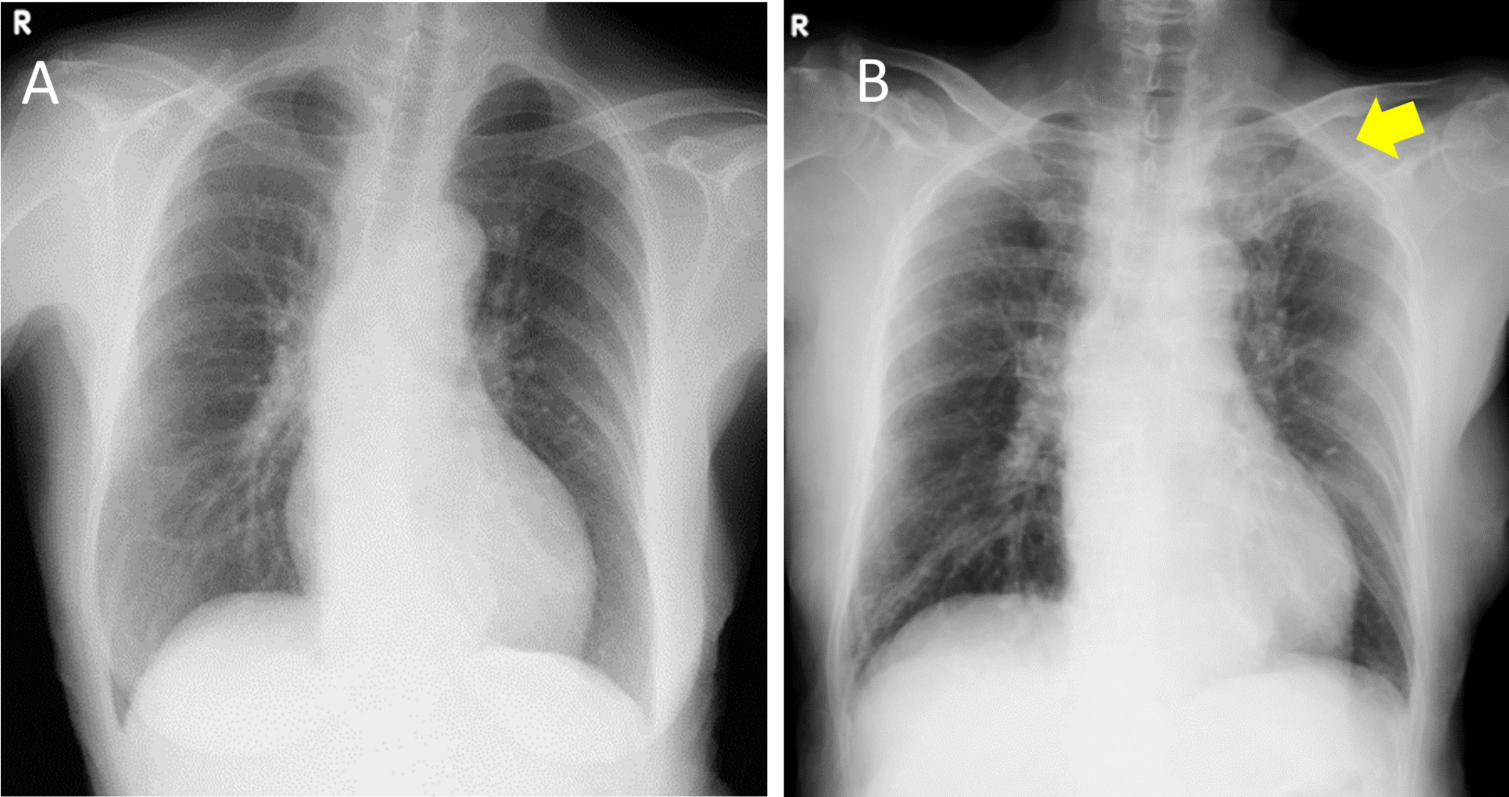
Chest Radiographs Before and After Pneumonia Onset. (A) Chest radiograph prior to the onset of pneumonia. (B) Chest radiograph following the onset of pneumonia, demonstrating decreased radiolucency in the left upper lung field (yellow arrow).

Chest radiography on initial evaluation revealed decreased radiolucency with infiltrates in the left upper lung field (Figure 1).

**Figure 2:**
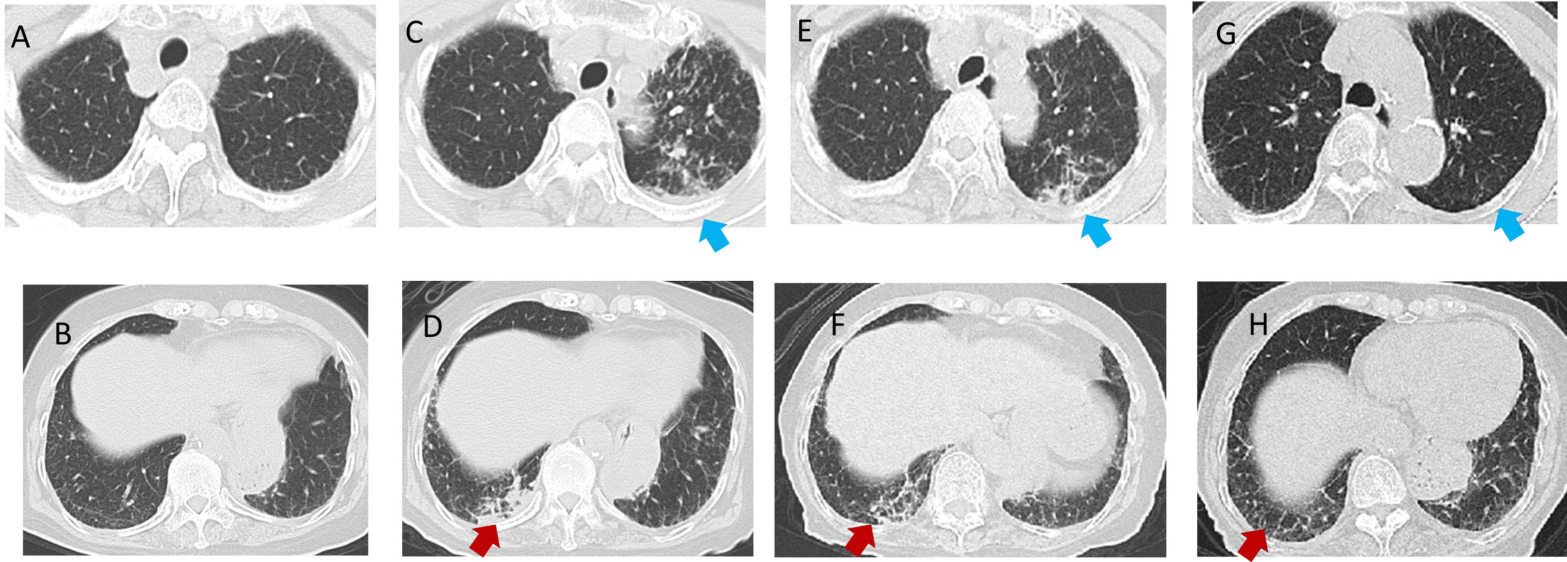
Serial Chest CT Scans Prior to Onset, at Onset, and During Treatment. (A, B) CT images were obtained before the onset of pneumonia. (C, D) CT on X month Y day (at the time of referral): granular opacities in the left upper lobe (blue arrows) and patchy opacities in the right lower lobe (red arrows) are evident. (E, F) CT on X month Y+14 day: both findings persist. (G, H) CT on X+6 months: opacities in both lobes have resolved. CT: computed tomography

**Figure 3:**
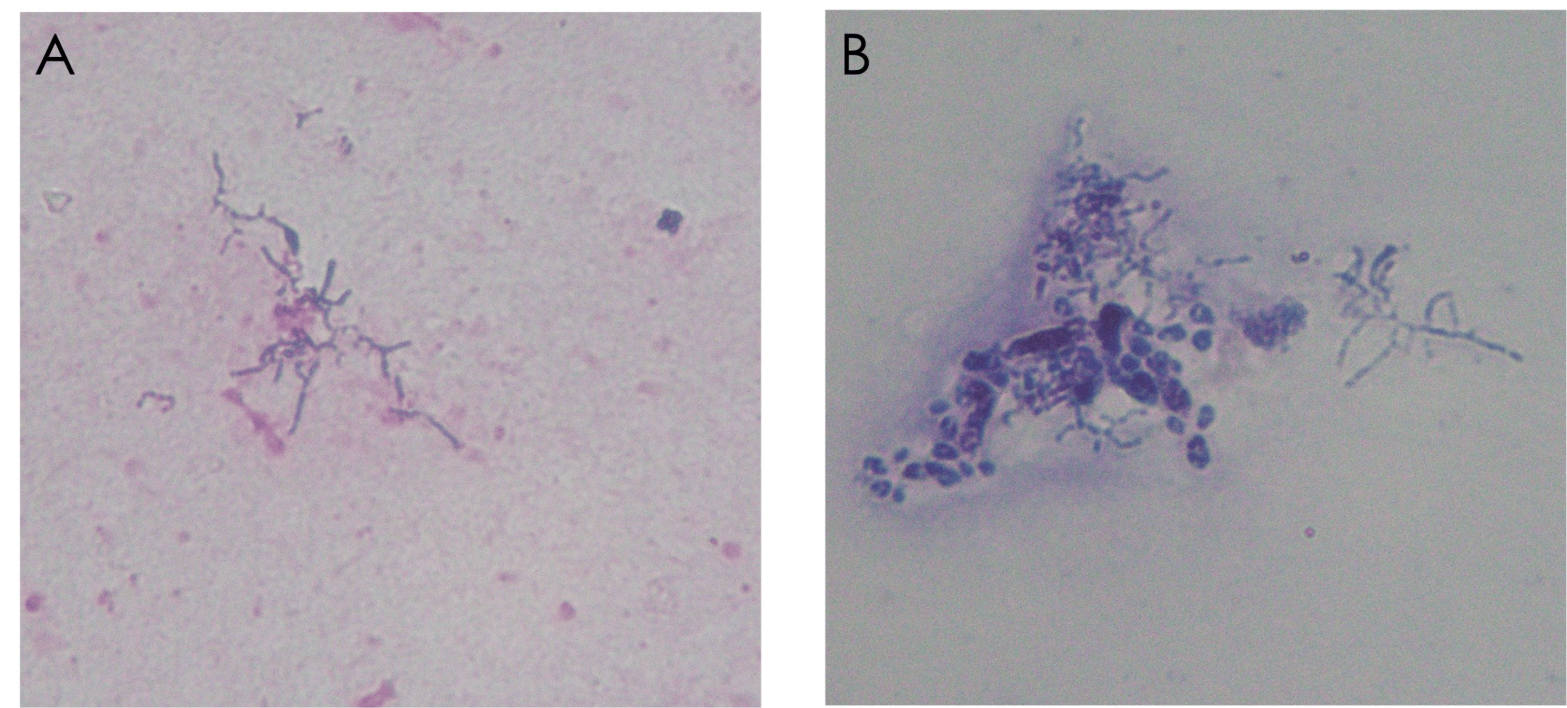
Gram Staining and Kinyoun Staining of Sputum Sample Showing Rothia aeria. (A) Gram staining (×1000, oil immersion) revealed branched gram-positive rods. (B) Kinyoun staining (×1000, oil immersion) was performed to evaluate for *Nocardia* infection, but no acid-fast organisms were observed, and no evidence of phagocytosis was seen. The organism was subsequently identified as *Rothia aeria* by 16S ribosomal ribonucleic acid gene sequencing.

Chest computed tomography (CT) revealed centrilobular granular opacities in the left upper lobe and patchy infiltrates in the right lower lobe (Figure 2), which were consistent with typical lower lobe-dominant aspiration pneumonia. Although a formal swallowing assessment had not been performed, the patient reported postprandial coughing. Based on the clinical setting of prolonged bed rest with supine feeding, postprandial coughing, and bilateral lower lobe-predominant infiltrates, typical of aspiration pneumonia, the initial diagnosis was made as aspiration pneumonia. The absence of other typical features of atypical or hematogenous pneumonia further supported this impression. The sputum sample submitted on day Y was evaluated as Geckler Group 5, and Gram staining revealed branching Gram-positive bacilli (Figure 3).

Based on the Gram stain findings, *Nocardia* and actinomycosis were considered in the differential diagnosis. However, given the clinical context of aspiration pneumonia and the need for broad-spectrum coverage, including anaerobes and Gram-negative bacilli, empirical treatment with meropenem (0.5 g every 12 hours) was initiated. Although trimethoprim-sulfamethoxazole (TMP-SMX) and doxycycline are effective against *Nocardia*, they were not chosen initially. After 16S rRNA sequencing identified the organism as *R. aeria*, meropenem was continued due to ongoing clinical and radiological improvement, and its known susceptibility profile. CRP levels rapidly improved, and her clinical symptoms resolved.

Kinyoun staining was performed to evaluate for *Nocardia* infection, but was negative, and no phagocytosed organisms were observed (Figure 3). 16S rRNA sequencing was performed on the sputum culture specimen, which identified the organism as* R. aeria*.

Antimicrobial susceptibility testing was performed using the breakpoint interpretation criteria for *Staphylococcus* species, as no specific standards exist for *Rothia* species. The isolate was interpreted to be susceptible to penicillins, cephalosporins, quinolones, and carbapenems. On day Y+13, *R. aeria* was again isolated from a high-quality sputum specimen. Although this raised concerns about persistent infection, the patient remained clinically stable, and inflammatory markers were improving. To evaluate the necessity of continuing antibiotics, a bronchoscopy was deliberately performed on day Y+14, prior to discontinuing meropenem. No organisms were detected in the BAL fluid. We interpreted the positive sputum culture as residual colonization or non-viable bacteria under ongoing antimicrobial pressure. In the absence of radiological or clinical deterioration, meropenem was discontinued, and no recurrence was observed during follow-up. The close timing was intentional to evaluate for persistent lower airway infection before stopping antimicrobial therapy. Absence of pathogens in BAL and subsequent clinical stability supported the decision to discontinue antibiotics. By day Y+180 (approximately six months after the onset of pneumonia), follow-up imaging confirmed complete resolution of pulmonary infiltrates, and sputum cultures remained negative (Figure 2). Based on the clinical history and radiological findings, the initial diagnosis was aspiration pneumonia. However, given the repeated isolation of *R. aeria* from sputum and favorable response to antibiotics, the possibility of respiratory tract infection caused by this organism was considered.

## Discussion

*Rothia* species are facultatively aerobic Gram-positive bacilli belonging to the order Actinomycetales, similar to *Actinomyces *and *Nocardia *[1]. *R. aeria* was first isolated from the air in the Mir space station in 2004 [2] and has since been reported as an opportunistic pathogen causing infections such as pneumonia, bacteremia, arthritis, and endocarditis, primarily in immunocompromised patients [2-4]. In contrast, our patient was considered immunocompetent based on the absence of malignancy, immunosuppressive therapy, or hematologic disease. Although she had well-controlled hypertension and diabetes, there was no evidence of systemic immune dysfunction. However, its pathogenicity and optimal treatment duration remain unclear.

In the present case, *R. aeria* was isolated from sputum specimens on two separate occasions: the first on day Y (at the time of pneumonia diagnosis), and the second on day Y+13 (near the end of antimicrobial therapy). Both samples were Geckler Group 5, indicating good-quality sputum, and cultures were performed before and near the end of treatment, respectively. The Gram stain revealed branching bacilli, prompting suspicion of *Nocardia* or actinomycosis. However, Kinyoun staining was negative, phagocytosis was absent, and 16S rRNA sequencing identified the organism as *R. aeria*. A 14-day course of meropenem led to rapid clinical and radiographic improvement. Subsequent BAL cultures were negative, suggesting a limited infection. Although it is uncertain whether the improvement resulted from specific treatment against* R. aeria* or the antimicrobial effect against other undetected pathogens, repeated isolation, radiological worsening, absence of other pathogens, and favorable response support the interpretation of a mild, atypical infection rather than colonization. This case underscores the challenge of distinguishing colonization from infection when rare organisms are isolated from non-sterile specimens.

*R. aeria* is known to be susceptible to many antimicrobial agents, including penicillins, cephalosporins, carbapenems, macrolides, and TMP-SMX [3]. In this case, the organism showed a good response to meropenem. Most *Rothia *infections [5,6] reported in the literature have been mild and resolved with approximately two weeks of antibiotic therapy. To our knowledge, only four cases of *R. aeria* pneumonia have been reported (Table 2) [7-10].

**Table 2:** Summary of Reported Cases of Pneumonia Caused by Rothia aeria This table summarizes previously reported cases of *Rothia aeria*-associated pneumonia, including patient background, immune status, radiological findings, treatment regimens, and clinical outcomes. Immunocompromised status was defined based on the presence of malignancy, immunosuppressive therapy, or hematologic disorders. Treatment duration varied depending on disease severity and host condition. allo-HSCT: allogeneic hematopoietic stem cell transplantation; 16S rRNA sequencing: 16S ribosomal ribonucleic acid gene sequencing; MALDI-ToF-MS: matrix-assisted laser desorption/ionization time-of-flight mass spectrometry; AMPC: amoxicillin; CP: cefpodoxime; CVA: clavulanic acid; MFLX: moxifloxacin; PCG: benzylpenicillin; LVFX: levofloxacin; MCFG: micafungin; CPFX: ciprofloxacin; TMP-SMX: trimethoprim-sulfamethoxazole; MEPM: meropenem

S. No. & Reference	Age	Sex	Background	Medications Used	Diagnosis	Examination Method	Identification Method	Antibiotics Used	Duration of Treatment
1 [7]	66	Male	Rheumatoid arthritis	Etanercept	Acute bronchiolitis	Sputum	16S rRNA sequencing	AMPC + CP for 3 weeks; AMPC/CVA + MFLX for 1 week	4 weeks
2 [8]	53	Female	Neurosarcoidosis	Azathioprine, prednisolone	Cavitary pneumonia	Bronchoalveolar lavage fluid	16S rRNA sequencing	PCG for 3 months, followed by AMPC for 5 months	8 months
3 [9]	61	Male	Myelodysplastic syndrome (allo-HSCT)	Not specified	Bacterial pneumonia	Bronchoalveolar lavage fluid	MicroScan WalkAway system (Beckman Coulter)	LVFX + MCFG + AMPC/CVA + CPFX	At least 4 weeks (estimated)
4 [10]	80	Female	Dyslipidemia, hyperuricemia	Not specified	Bacterial pneumonia	Sputum	MALDI-ToF-MS	TMP-SMX, others unknown	2 weeks
This Case	80s	Female	Hypertension, diabetes	Not specified	Bacterial pneumonia	Sputum	16S rRNA sequencing	MEPM	2 weeks

Among these, three cases occurred in immunocompromised patients who required more than four weeks of antimicrobial therapy, while the remaining case, similar to ours, involved an immunocompetent patient who improved with a 14-day course. These findings highlight the variability of clinical presentation and the need for individualized treatment strategies based on host immunity and infection severity.

Although *R. aeria* belongs to a group of organisms that can cause serious infections, not all isolations require prolonged antibiotic treatment. In our case, clinical and radiological improvement was observed following a short course of meropenem, and post-treatment cultures were negative. While a direct therapeutic effect on *R. aeria* cannot be definitively confirmed, the repeated detection, temporal correlation with symptoms, absence of other pathogens, and rapid improvement suggest that this organism contributed to a transient, mild respiratory infection. The absence of significant inflammation or tissue destruction on Gram stain further supports this interpretation. This case also illustrates the diagnostic difficulty in determining the clinical relevance of rare organisms in non-sterile respiratory specimens. We acknowledge several limitations in interpreting *R. aeria* as the causative pathogen in this case. First, *R. aeria* was isolated only from non-sterile sputum specimens. Although a BAL was performed to assess for persistent infection, it was done after the completion of antibiotic therapy and yielded no organisms, limiting microbiological confirmation. Second, the patient was treated with a broad-spectrum antibiotic (meropenem), which may have led to clinical improvement by targeting undetected pathogens unrelated to *R. aeria*. These factors prevent definitive attribution of causality. Nonetheless, the repeated isolation of *R. aeria* from high-quality sputum, the absence of alternative pathogens, radiological worsening, and clinical response to therapy suggest that *R. aeria* may have contributed, at least partially, to the infectious process.

Importantly, the detection of *Rothia* species in respiratory specimens, especially in immunocompetent individuals, does not necessarily mandate long-term or aggressive antibiotic therapy. In mild cases, empirical treatment similar to that for community-acquired pneumonia may be sufficient. Given the clinical heterogeneity of *R. aeria *infections, therapeutic decisions should be tailored to the host immune status, clinical course, and response to treatment.

## Conclusions

This case illustrates a rare instance of transient respiratory detection of *R. aeria* in an immunocompetent elderly patient, with resolution achieved through a 14-day course of meropenem. Although distinguishing between infection and colonization was diagnostically difficult, the clinical course, imaging findings, and improvement following antibiotic therapy suggest that the repeated isolation of the organism most likely represented transient colonization. However, given the radiological progression and absence of other pathogens, a mild infectious component cannot be completely excluded. Importantly, meropenem was initiated empirically to target anaerobic and Gram-negative organisms due to suspected aspiration pneumonia. While* R. aeria *was not the primary target of therapy, it demonstrated susceptibility to meropenem. Therefore, the observed clinical response could have been due to the treatment of undetected pathogens, and this limitation should be acknowledged when interpreting causality.

*Rothia* species are typically susceptible to common antibiotics, and many reported cases have responded well to short-term therapy. Accordingly, detection of *R. aeria* in respiratory specimens does not necessarily warrant prolonged antibiotic administration. Comprehensive clinical judgment integrating microbiological, radiological, and therapeutic response is essential in determining the significance of rare organisms in non-sterile specimens.
